# The relationship of caregiver strain with resilience and hardiness in family caregivers of older adults with chronic disease: a cross-sectional study

**DOI:** 10.1186/s12912-022-00966-3

**Published:** 2022-07-11

**Authors:** Ashkan Sorayyanezhad, Nasrin Nikpeyma, Shima Nazari, Farshad Sharifi, Naeimeh Sarkhani

**Affiliations:** 1 Department of Community Health and Geriatric Nursing, School of Nursing and Midwifery, Tehran University of Medical Sciences, Tehran, 1419732171 Iran; 2 Department of Psychiatric Nursing, School of Nursing and Midwifery, Tehran University of Medical Sciences, Tehran, Iran; 3 Elderly Health Research Center, Endocrine Population Sciences Institute, Endocrinology, and Metabolism Research Institute, Tehran University of Medical Sciences, Tehran, Iran; 4 Nursing and Midwifery Care Research Center, School of Nursing and Midwifery, Tehran University of Medical Sciences, Tehran, Iran

**Keywords:** Caregiver, Caregiver strain, Resilience, Hardiness, Older adults

## Abstract

**Background:**

Providing long-term home care to older adults with chronic diseases may endanger the physical, mental, social, and spiritual health of caregivers and lead to care strain.

**Objective:**

This study aimed to assess the relationship of caregiver strain with resilience and hardiness in family caregivers of older adults with chronic disease.

**Methodology:**

This cross-sectional correlational study was conducted in 2020–2021 in Tehran. Participants were 230 family caregivers randomly recruited from 8 urban health care centers. Data were collected using a personal characteristics questionnaire, the Modified Caregiver Strain Index, Connor-Davidson Resilience Scale, Family Hardiness Index, and the data were analyzed by using SPSS 22 version. Pearson’s correlation coefficient was applied for data analysis. *P*-values ≤0.05 were considered significant.

**Results:**

A total of 230 caregivers participated in the study. The mean age of participants was (46.65 ± 13.63) years and most of them were female (73.9%). Mean scores of caregiver strain, resilience, and hardiness in family caregivers were 16.23 ± 4.5, 39.89 ± 10.9, and 31.21 ± 7.79, respectively. Pearson correlation showed a significant and inverse correlation between caregiver strain and resilience (*r* = -0.310, *P* = 0.002), and also a significant and inverse relationship between caregiver strain and hardiness (*r* = -0.276, *P* = 0.001).

**Conclusion:**

In this study, family caregivers had moderate caregiver strain, low resilience, and high hardness. Caregiver strain in family caregivers of older adults with chronic disease is an important health issue associated with resilience and hardiness. To promote health, effective adaptation to long-term care, and reduce caregiver strain, designing effective interventions to increase resilience and hardiness in family caregivers seems necessary.

## Introduction

The aging population of the world has become a health and care challenge. The elderly population of Iran is estimated to reach 26 million by 2050, which is 26% of the total population of the country [1]. Increasing the number of older adults and life expectancy is associated with health problems, one or more chronic diseases, and disability [2]. Older adults with chronic illness need long-term support and care, and both themselves and their family members and other dependents are affected [3]. With the increase in hospitalization costs and the willingness of the older adults to stay with their spouse and children, care for the elderly is often provided by a family caregiver [4]. Although family care has positive effects on the patient and the caregiver is aware of the patient’s needs and habits and can better address these needs, this type of care affects different aspects of caregiver life [5] and facilitates or Difficulty in balancing the different roles and activities of caregivers’ lives and how to care for the elderly [6].

Older adults care can have unpleasant consequences for various aspects of health, quality of life, social relationships, and financial conditions of caregivers [7]. One of the most important consequences of care is the caregiver strain (CS). Caregiver strain can be defined as “the level of multifaceted strain perceived (objective or subjective) by the caregiver from caring for a family member and/or loved one over time”. Caregiver strain is a condition that seeks to endure the problems and difficulties of care for caregivers and leads to negative effects on various aspects of health [8]. In a community-based survey, Koyanagi et al. reported that family caregivers are under the stress of caring, while caregiver strain is also linked to factors such as gender, education, care hours, depression, and financial problems [9]. Instead of focusing on the nature of the caregiver strain, new perspectives emphasize the role of the individual’s psychological resources in dealing with caregiver strain factors. Resilience is one of the psychological components and various definitions of resilience are found in the scientific literature. According to the American Psychological Association, resilience is the process and outcome of successfully adapting to difficult or challenging life experiences, especially through mental, emotional, and behavioral flexibility and adjustment to external and internal demands. Resilience in caregivers means the ability of caregivers to adapt positively to difficulties and injuries, which is shown after facing the pressure and stress caused by long-term care [10]. Resilience increases self-care and increases a person’s ability to deal with problems related to caring for older adults. Resilient individuals adapt to environmental changes and return to recover quickly after the stressors have resolved [11]. The results of studies have shown that resilience in patients’ family caregivers is associated with less depression, increased health, positive social support, and improved quality of care for older adults [12–14].

Another important psychological component in coping with difficult situations, problem-solving, and stress management is hardiness [1]. Hardiness is defined as a skill that prepares a person to face life’s problems. Hardiness has three main components, which include control (the ability to control different life situations), commitment (willingness to participate), and challenge (the ability to understand that changes in life are normal). Hard people feel more committed and in control of their work and see problems as potential opportunities for change [15]. Hardiness and coping styles Influence the perception of stress and people’s performance in stressful situations. The relationship between hardiness and coping styles has been confirmed [16]. The results of various studies have shown that hardiness has significant effects on the quality of life of older adults and the mental health of caregivers [1, 17].

Maintaining health and the need to identify factors affecting the vulnerability is important for family caregivers who have been providing care for older adults for a long time and are also emotionally dependent on them. Considering that the components of resilience and hardiness have an effect on promoting health and increasing the quality of life and due to the scarcity of studies related to the relationship of caregiver strain with resilience and hardiness in family caregivers of older adults with chronic disease, this study was conducted.

### Questions

Is there an association between caregiver strain with resilience in family caregivers of older adults with chronic disease? Is there an association between caregiver strain with hardiness in family caregivers of older adults with chronic disease?

## Methods

### Design

This cross-sectional correlational study was conducted in 2020–2021 in urban health care centers affiliated with Tehran University of Medical Sciences in Iran.

### Setting and sample

The study population comprised family caregivers of older adults with chronic diseases, who had records in the urban health care centers. Inclusion criteria for the participants were: an age of 18 years or older, family caregivers (spouse, children, relatives or friends), providing care of older adults with chronic diseases and score less than 4 based on Katz’ Activities of Daily Living instrument, no known mental disorders, and providing care to older adults for 21 hours per week.

With a confidence level of 95%, a power of 80%, and a probable attrition rate of 40%, the error of the first type (α) 0.05 and the error of the second type (β (0.10, and considering the Spearman correlation coefficient based on the study of Hsiao et al., 230 samples were calculated [18]. Sampling was performed by multi-stage method. Out of 33 urban health care centers affiliated with Tehran University of Medical Sciences located in 5 areas of the south of Tehran, 8 centers were randomly selected (lottery), and then, by reviewing the files of the older adults and identifying their caregivers, 230 eligible caregivers from equally selected centers (29 samples from each center) were randomly selected based on Rand random number table.

#### Data collection

Data were collected using a personal characteristics questionnaire, the Activities of Daily Living (ADL), Caregiver Strain Index (CSI), Connor-Davidson Resilience Scale (CD-RISC), Family Hardiness Index (FHI). Participants were asked to complete these instruments through self-report.

Items included on the personal characteristics questionnaire were age, gender, marital status, number of children, education, duration of care, income, relationship with older adults, history of drug use, history of surgery, insurance, and living status.

The Activities of Daily Living (ADL) instrument developed by Katz et al. [19] includes 6 items on a two-way scale from 0 (dependent) and 1 (independent), resulting in a total score of 0 to 6, with a higher score than 4 indicating relative independence, and a lower score than 2 indicating strong dependence. The Intra Class Correlation (ICC) coefficient of this scale was reported to be 0.82. This instrument was translated into Persian and psychometrically evaluated in a previous study in Iran, which reported Cronbach’s alpha and ICC coefficient to be more than 0.75 [20].

The Modified Caregiver Strain Index (MCSI) developed by Robinson (1983) includes 13 items scored on a 3-point scale from 0 (never) to 2 (always), resulting in a total score of 0 to 26, with higher scores indicating greater caregiver strain. The internal consistency and the Intra Class Correlation (ICC) coefficients of this scale were reported to be 0.86 and 0.88, respectively [21, 22]. This scale was translated into Persian and psychometrically evaluated in a previous study in Iran, which reported a Cronbach’s alpha of 0.81 [23].

The Connor-Davidson Resilience Scale (CD-RISC) includes 25 items with the perception of individual competence (8 items), trust in individual instincts, tolerance of negative emotions (7 items), positive acceptance of change, and safe relationships (5 items), control, and spiritual effects (5 items). Items are scored on a 5-point Likert scale from 0 (true) to 4 (completely false), resulting in a total score of 0 to 100. The cut-off point of the scale is 50, with higher scores than 50 indicating greater resilience. The internal consistency and the Intra Class Correlation (ICC) coefficients of this scale were reported to be 0.89 and 0.87, respectively [24]. This scale was translated into Persian and psychometrically evaluated in a previous study in Iran, which reported a Cronbach’s alpha of 0.87 [25].

The Family Hardiness Index (FHI) developed by McCubbin et al. (1996) includes 20 items with the commitment (8 items), challenge (6 items), and control (6 items). Items are scored on a 4-point Likert scale from 0 (completely wrong) to 3 (completely correct), resulting in a total score of 0 to 100. The cut-off point of the scale is 50, with higher scores than 30 indicating greater hardiness [26]. This scale was translated into Persian and psychometrically evaluated in a previous study in Iran which reported a Cronbach’s alpha of 0.84, 0.73, 0.64, and 0.76 for the subscales, respectively [27].

### Ethical considerations

After obtaining permission to conduct research, eligible samples were selected from the electronic files of older adults in the selected urban health care centers and their families. The objectives of the research were explained to them and it was ensured that participation in the research was voluntary and that their information would remain confidential. Written informed consent was obtained from all participants, and then the self-report scales were completed by caregivers in 30–40 minutes in the research settings.

### Statistical analyses

Descriptive statistics were calculated for the personal characteristics of the participants. Categorical variables were described using frequencies and percentages. Mean and standard deviation was used for the description of care strain, resilience, and hardiness. The distribution of data was normal with Kolmogorov–Smirnov test. Pearson correlation coefficient was used to determine the correlation between CS and resilience, and caregiver CS and Hardiness. The *P*-values were ≤ 0.05. Data were analyzed using SPSS version 22.

### Findings

In total, 230 sets of instruments were distributed among 230 participants. All participants completely answered the study instruments and all were included in the final analysis. The mean age of caregivers of older adults was 46.65 ± 13.63 years. Most participants were female (73.9%), and lived with older adults (59.1%) (Table 1).

**Table 1:** Frequency distribution of personal characteristics in Family caregivers of Older Adults with Chronic disease

Personal characteristics	Number (abundance)	percentage
**Gender**		
Male	60	26.1
Female	170	73.9
**Marital status**		
Single	50	17.8
Married	166	72.2
widow	4	1.7
divorced	10	4.3
**Number of children**		
Non	73	31.7
1–2	90	39.1
3-4	56	24.3
5 and more	11	4.8
**Education**		
Illiterate	33	14.3
High school	108	47
Graduated from high school	46	20
bachelor	42	18.3
Masters- Ph.D.	1	0.4
**Duration of care**		
12 months	59	25.7
13-24 month	63	27.2
25-60 month	65	28.1
More than 60 months	43	19
**Income**		
Enough	18	7.8
Almost enough	124	53.9
Not enough	88	38.3
**Relationship with older adult**		
spouse	125	54.3
child	76	33
Friend and relatives	29	12.7
**History of drug use**		
yes	74	32.2
no	156	67.8
**History of surgery**		
yes	60	26.1
no	170	73.9
**Insurance**		
have	205	89.1
Not have	25	10.9
**Living status**		
With older adult	136	59.1
In another place	94	40.9
Mean of age ± Std	46.65 ± 13.63	

The mean scores of participants’ Cs (range 0–26) were 16.23 ± 4.5. Caregivers experienced the highest and lowest CS in the physical 4.78 ± 1.98 and the personal areas 2.3 ± 1.5, respectively.

The mean scores of participants’ resilience (range of 0–100) were 39.89 ± 10.9. The highest and lowest resilience scores were 14.50 ± 8.98 in the dimension of trust in individual instincts and 7.56 ± 3.95 in the dimension of control of spiritual effects, respectively (Table 2). The mean scores of participants’ hardiness (range of 0–60) were 31.21 ± 7.79, the highest and lowest hardiness scores were in the commitment 19.78 ± 13.4, and the control dimension 17.27 ± 13.45, respectively (Table 3).

**Table 2:** Correlation between CS and resilience in Family caregivers of Older Adults with Chronic disease

resilience	Perception of individual competence	Trust in individual instincts	positive acceptance of change, and safe relationships	Control and spiritual effects	Total resilience	Mean ± Std
caregiver strain						
**physical**	-0.210 (*P* = 0/56)	-0.198 (*P* = 0/001)	-0.201 (*P* = 0/03)	-0.145 (*P* = 0/001)	-0.216 (*P* = 0/001)	4.78 ± 1.98
**psychological**	-0.219 (*P* = 0/67)	-0.289 (*P* = 0/01)	-0.200 (*P* = 0/98)	-0.219 (*P* = 0/015)	-0.223 (*P* = 0/001)	3.5 ± 2.15
**social**	-0.216 (*P* = 0/001)	-0.301 (*P* = 0/123)	-0.267 (*P* = 0/401)	-0.323 (*P* = 0/18)	-0.287 (*P* = 0/001)	2.24 ± 1.54
**personal**	-0.321 (*P* = 0/001)	-0.289 (*P* = 0/01)	-0.210 (*P* = 0/145)	-0.321 (*P* = 0/002)	-0.298 (*P* = 0/08)	2.3 ± 1.5
**economical**	-0.156 (*P* = 0/003)	-0.087 (*P* = 0/001)	-0.219 (*P* = 0/118)	-0.305 (*P* = 0/21)	-0.290 (*P* = 0/001)	2.8 ± 1.88
**Total caregiver strain**	-0.421 (*P* = 0/001)	-0.321 (*P* = 0/02)	-0.218 (*P* = 0/13)	-0.132 (*P* = 0/12)	-0.310 (*P* = 0/002)	16.23 ± 4.5
**Mean ± Std**	12.78 ± 9.1	14.50 ± 8.98	10.27 ± 5.13	7.56 ± 3.95	39.89 ± 10.9

**Table 3:** Correlation between CS and family hardiness in Family caregivers of Older Adults with Chronic disease

Family hardness	Commitment	Challenge	Control	Total family hardiness
Caregiver strain				
**Physical**	-0.110 (*P* = 0/06)	-0.189 (*P* = 0/005)	-0.186 (*P* = 0/04)	-0.187 (*P* = 0/001)
**Psychological**	-0.119 (*P* = 0/167)	-0.079 (*P* = 0/01)	-0.123 (*P* = 0/18)	-0.118 (*P* = 0/012)
**Social**	-0.076 (*P* = 0/001)	-0.201 (*P* = 0/433)	-0.189 (*P* = 0/399)	-0.010 (*P* = 0/001)
**Personal**	-0.121 (*P* = 0/002)	-0.101 (*P* = 0/001)	-0.176 (*P* = 0/175)	-0.121 (*P* = 0/156)
**Economical**	-0.032 *P* = (0/001)	-0.047 *P* = (0/001)	-0.119 (*P* = 0/318)	-0.032 (*P* = 0/001)
**Total caregiver strain**	-0.032 (*P* = 0/001)	-0.121 (*P* = 0/02)	-0.118 (*P* = 0/23)	-0.076 (*P* = 0/001)
**Mean ± Std**	19.78 ± 13.4	18.5 ± 12.28	17.27 ± 13.45	31.21 ± 7.79

Pearson correlation coefficients between CS and resilience of participants were obtained (*r* = -0.310). The correlation between the total resilience score and CS subscales was significant and inverse (*P* = 0.002) (Table 2). Also, the Pearson correlation coefficient between CS and the hardiness of participants was reported (*r* = -0.076). Except in the personal subscale, the correlation between total hardiness score and CS subscales was significant and inverse (*P* = 0.001) (Table 3).

## Discussion

The study aimed to assess the relationship of caregiver strain with resilience and hardiness in family caregivers of older adults with chronic diseases. The results of this study showed that caregivers of older adults with chronic caregiver strain disease experienced more than average. Shafiezadeh et al. found that caregiver strain was higher than average in family caregivers of older adults with Alzheimer’s disease [28]. Also, in a study in the United States, 45.9% of caregivers reported moderate caregiver strain [8], which is consistent with the results of the present study [28]. .Also, in a study in the United States, 45.9% of caregivers reported moderate caregiver strain [8], which is consistent with the results of the present study. Zhang et al. in a study using the CSI found that more than 50% of caregivers of dementia patients experienced high levels of caregiver strain, which is not consistent with the present study [29].

In this study, caregivers experienced the most physical injury and the least personal injury. Elderly people with chronic diseases, due to dependence and limitation in their activity, have difficulty in achieving their needs and most of their responsibility is the responsibility of caregivers, so they have experienced the highest physical injury.

In this study, caregivers experienced the most physical injury and the least personal injury. Older adults with chronic disease have difficulty meeting their needs due to dependence on others and limited activity and appear to be the cause of more physical care strain. Marmol et al. Reported that family caregiver strain is related to dependence on daily life activities and cognitive status of hospitalized patients [30]. Caregivers received the lowest caregiver strain in the personal domain. It can be said that 71% of caregivers in this study had 3 to 4 children and were likely to assist caregivers in care providing to older adults, so that family caregivers could take care of their personal activities.

The findings of this study showed that the resilience of family caregivers was low, which is consistent with the studies of Vagharseyyedin et al. And Meikaeilei et al. [31, 32]. Ong et al. reported moderate resilience in caregivers of older adults with chronic disease in Singapore, using a CD-RISC in this study as in the present [33]. The difference between the Ong et al. study and the present study could be related to the quality of social and psychological services provided to family caregivers in Singapore, the culture, and the implementation of psychological training programs and social support plans for family caregivers. In another study, resilience was reported to be moderate in family caregivers of patients with mental disorders [34]. The difference between the results of resilience in these two studies can be due to the difficult situation of providing care to older adults, their dependence on daily work, and the burden of care on family caregivers. In addition, the average age of patients with mental disorders was lower and they were independent in daily activities. Resilience is the result of trying to achieve balance and proper functioning in stressful and critical situations. Previous unresolved problems, changes in the family life cycle, and the presence of a family member with a chronic disease increase family expectations and needs, resulting in reduced resilience in caregivers [35].

Findings of this study showed that hardiness was high in caregivers of older adults with chronic disease, which is consistent with the study by Eyni & Hashemi [1]. Asli et al. reported low hardiness in caregivers of people with physical-mental disabilities which is not consistent with the present study [17]. This difference can be in the type of instruments used, the research community, the difficult conditions of providing care to people with disabilities, and more psychological strain than providing care to older people. Hardiness as an individual trait, makes people feel better about themselves and the world around them and feel more satisfied and successful in life. In fact, hard people have a pattern of commitment, control, and struggle, and these patterns make people more effective in the face of adversity.

The results of the present study showed that there is a statistically significant and inverse relationship between caregiver strain and resilience and hardiness in family caregivers. As the caregiver strain increases, resilience and hardiness decrease, and vice versa. Chan et al. reported that there was a significant inverse relationship between care strain and resilience in family caregivers of older adults with Alzheimer’s disease [36]. A similar study showed that with increasing resilience in caregivers of dementia patients, their caregiver strain decreases [37]. Dias et al. found that psychological factors were associated with high resilience. Thus, social support was a moderator of resilience, and social support was useful to reduce physical and psychological strain and increase resilience [38]. The results of a study showed that there is a significant and inverse relationship between hardiness and caring pressure in caregivers of older people with schizophrenia. The higher the hardiness, the lower the care pressure on caregivers and vice versa [39]. Due to cultural conditions in most parts of Iran, family care for older adults is an integral part of life that is done with kindness and empathy. In addition, formal support facilities for older people are limited, which has led families to provide care to older people as family caregivers. Therefore, policies should focus on interventions to empower family caregivers.

## Limitations

The present study had limitations that challenge the generalizability of the findings. This study was performed only on older adult caregivers in a city in Iran. Repeating similar studies on larger samples of family caregivers of older people is suggested in different areas. The tendency to respond positively and negatively (social desirability bias) is one of the possible biases in completing research tools that may also be discussed in this research. Therefore, it is recommended to use other methods of data collection such as interviewing and observation to obtain more accurate information.

## Conclusion

The findings of this study showed that family caregivers of older adults with chronic disease experience moderate to high levels of caregiver strain. The findings of this study show the importance of the relationship between caregiver strain and hardiness and resilience and can be useful in designing interventions to increase resilience and hardiness and reduce caregiver strain in family caregivers of older adults with chronic disease. Nurses can play an important role in screening caregivers’ health and promoting resilience and hardiness, Therefore, it is recommended that nurses provide the necessary training on mental and physical health to Family caregivers of older adults. Also, it is recommended that planning be done to support caregivers through health policymakers.

## Data Availability

The datasets generated and/or analyzed during the current study are not publicly available due to keep participants’ information confidential, but are available from the corresponding author on reasonable request.

## References

[1] Hashemi Z, Eyni S (2020). Perceived stress in the elderly: the role of spiritual intelligence, self-compassion, and psychological hardiness. Aging Psychol.

[2] Botes R, Vermeulen KM, Correia J, Buskens E, Janssen F (2018). Relative contribution of various chronic diseases and multi-morbidity to potential disability among Dutch elderly. BMC Health Serv Res.

[3] Schenker M, Costa DH (2019). Advances and challenges of health care of the elderly population with chronic diseases in primary health care. Cien Saude Colet.

[4] Aboozadeh Gotabi K, Ghanbari Moghaddam A, Mohammadi M, Zarei F (2016). The burden of family caregivers caring for older adults and its relationship with some factors. Nurs Vulnerable J.

[5] Coe NB, Guo J, Konetzka RT, Van Houtven CH (2019). What is the marginal benefit of payment-induced family care? Impact on Medicaid spending and health of care recipients. Health Econ.

[6] Camicia M, Lutz BJ, Markoff N, Catlin A (2019). Determining the needs of family caregivers of stroke patients during inpatient rehabilitation using interview, art, and survey. Rehabil Nurs J.

[7] National Academies of Sciences, Engineering, and Medicine (2016). Families caring for an aging America.

[8] King A, Ringel JB, Safford MM, Riffin C, Adelman R, Roth DL, Sterling MR (2021). Association between caregiver strain and self-care among caregivers with diabetes. JAMA Netw Open.

[9] Koyanagi A, DeVylder JE, Stubbs B, Carvalho AF, Veronese N, Haro JM, Santini ZI (2018). Depression, sleep problems, and perceived stress among informal caregivers in 58 low-, middle-, and high-income countries: a cross-sectional analysis of community-based surveys. J Psychiatr Res.

[10] American Psychological Association. Resilience. Available from: https://www.apa.org/topics/resilience. Last accessed on 5 Apr 2020.

[11] Van Roij J, Brom L, Sommeijer D, van de Poll-Franse L, Raijmakers N (2021). Self-care, resilience, and caregiver burden in relatives of patients with advanced cancer: results from the eQuiPe study. Support Care Cancer.

[12] Hwang IC, Kim YS, Lee YJ, Choi YS, Hwang SW, Kim HM, Koh SJ (2018). Factors associated with caregivers’ resilience in a terminal cancer care setting. Am J Hosp Palliat Med.

[13] Palacio C, Krikorian A, Limonero JT (2018). The influence of psychological factors on the burden of caregivers of patients with advanced cancer: resiliency and caregiver burden. Palliat Support Care.

[14] Ghaffari F, Rostami M, Fotokian Z, Hajiahmadi M. Effectiveness of resilience education in the mental health of family caregivers of elderly patients with alzheimer’s disease. Iran J Psychiatry Behav Sci. 2019;13(3). 10.5812/ijpbs.69507.

[15] Ashoori J (2016). Investigation the role of social capital, social support and psychological hardiness in predicting life quality of elderly women. Q J Geriatr Nurs.

[16] Hsieh CJ, Hsieh HY, Chen PH, Hsiao YL, Lee S (2004). The relationship between hardiness, coping strategies and burnout in psychiatric nurses. Hu Li Za Zhi.

[17] Asli Azad M, Rajaei R, Farhadi T, Aghasi A, Shahidi L (2016). Investigating the relationship between hardiness as well as resiliency and burnout aspects in the care givers of the physically, mentally and multiple retarded patients at the welfare organization in 2015. Commun Health J.

[18] Hsiao CY, Tsai YF (2015). Factors of caregiver burden and family functioning among Taiwanese family caregivers living with schizophrenia. J Clin Nurs.

[19] Katz S, Downs TD, Cash HR, Grotz RC (1970). Progress in development of the index of ADL. The Gerontologist.

[20] Taheri Tanjani P, Azadbakht M (2016). Psychometric properties of the Persian version of the activities of daily living scale and instrumental activities of daily living scale in elderly. J Mazandaran Univ Med Sci.

[21] Robinson BC (1983). Validation of a caregiver strain index. J Gerontol.

[22] Thornton M, Travis SS (2003). Analysis of the reliability of the modified caregiver strain index. J Gerontol Ser B Psychol Sci Soc Sci.

[23] Ansari F (2016). The Relationship between burden of stroke and burden of caregiver with quality of life in survivors of stroke patients and their care-givers in clinics of Tabriz university of medical sciences.

[24] Connor KM, Davidson JR (2003). Development of a new resilience scale: the Connor-Davidson resilience scale (CD-RISC). Depress Anxiety.

[25] Samani S, Jokar B, Sahragard N (2007). Effects of resilience on mental health and life satisfaction. Iran J Psychiatry Clin Psychol.

[26] McCubbin MA, McCubbin HI, Thompson AI, Mccubbin y M, McCubbin H, Thompson A (1986). Family hardiness index (FHI). (1996), Family assessment: resiliency, coping and adaptation-Inventories for research and practice.

[27] Seyedi MS, Falah Tafti A, Sadeghi MS, Rezai K (2014). Role of attachment style and marital adaptation in family hardiness. Q J Health Breeze.

[28] Shafiezadeh A, Heravi-Karimoo M, Rejeh N, Sharif Nia H, Montazeri A (2019). Translation and primarily validation of the Persian version of caregiver burden inventory. Payesh (Health Monitor).

[29] Zhang M, Chang YP, Liu YJ, Gao L, Porock D (2018). Burden and strain among familial caregivers of patients with dementia in China. Issues Ment Health Nurs.

[30] Perez Marmol JM, Flores Antigüedad ML, Castro Sanchez AM, Tapia Haro RM, Garcia Rios MD, Aguilar Ferrandiz ME (2018). Inpatient dependency in activities of daily living predicts informal caregiver strain: A cross-sectional study. J Clin Nurs.

[31] Vagharseyyedin SA, Molazem Z (2013). Burden, resilience, and happiness in family caregivers of spinal cord injured patients. Middle East J Fam Med.

[32] Meikaeilei N, Ganji M, Talebi JM (2012). A comparison of resiliency, marital satisfaction and mental health in parents of children with learning disabilities and normal children. J Learn Disabil.

[33] Ong HL, Vaingankar JA, Abdin E, Sambasivam R, Fauziana R, Tan ME, Chong SA, Goveas RR, Chiam PC, Subramaniam M (2018). Resilience and burden in caregivers of older adults: moderating and mediating effects of perceived social support. BMC Psychiatry.

[34] Seyedfatemi N, Noghani F, Amini E, Kamali R (2018). Resiliency family caregivers of people with mental disorders in Tehran. Iranian. J Nurs Res.

[35] Mulud ZA, McCarthy G (2017). Caregiver burden among caregivers of individuals with severe mental illness: testing the moderation and mediation models of resilience. Arch Psychiatr Nurs.

[36] Chan EW, Yap PS, Khalaf ZF (2019). Factors associated with high strain in caregivers of Alzheimer's disease (AD) in Malaysia. Geriatr Nurs.

[37] Issari P, Tsaliki C (2017). Stories of family caregivers of people with dementia in Greece: implications for counselling. Eur J Psychother Counsell.

[38] Dias R, Santos RL, Sousa MF, Nogueira MM, Torres B, Belfort T, Dourado MC (2015). Resilience of caregivers of people with dementia: a systematic review of biological and psychosocial determinants. Trends Psychiatry Psychother.

[39] Abotalebi. The Association between Psychological Hardiness and Metacognition with Burden of Care in Caregivers of Patients with Schizophrenia, Nursing School in Sari. 2019. Doctoral dissertation.

